# NIPS workshop on New Problems and Methods in Computational Biology

**DOI:** 10.1186/1471-2105-8-S10-S1

**Published:** 2007-12-21

**Authors:** Gal Chechik, Christina Leslie, William Stafford Noble, Gunnar Rätsch, Quaid Morris, Koji Tsuda

**Affiliations:** 1 Computer Science Department, Stanford University, 353 Serra Mall, Stanford University, Stanford, CA 94305, USA; 2 Computational Biology Program, Memorial Sloan-Kettering Cancer Center, 1275 York Ave, Box 460, New York, NY 10065, USA; 3 Department of Genome Sciences, University of Washington, 1705 NE Pacific St, Seattle, WA 98109, USA; 4 Friedrich Miescher Laboratory of the Max Planck Society, Spemannstr. 39, 72076 Tübingen, Germany; 5 Terrence Donnelley Centre for Cellular and Biomolecular Research, University of Toronto, 160 College Street, Toronto, Ontario, M5S 3E1, Canada; 6 Max Planck Institute for Biological Cybernetics, Spemannstr. 38, 72076 Tübingen, Germany

December 8, 2006, Whistler, British Columbia, Canada

The field of computational biology has seen dramatic growth over the past few years, both in terms of available data, scientific questions and challenges for learning and inference. These new types of scientific and clinical problems require the development of novel supervised and unsupervised learning approaches. In particular, the field is characterized by a diversity of heterogeneous data. The human genome sequence is accompanied by real-valued gene expression data, functional annotation of genes, genotyping information, a graph of interacting proteins, a set of equations describing the dynamics of a system, localization of proteins in a cell, a phylogenetic tree relating species, natural language text in the form of papers describing experiments, partial models that provide priors, and numerous other data sources.

This supplementary issue consists of seven peer-reviewed papers based on the NIPS Workshop on New Problems and Methods in Computational Biology held at Whistler, British Columbia, Canada on December 8, 2006. The Neural Information Processing Systems Conference is the premier scientific meeting on neural computation, with session topics spanning artificial intelligence, learning theory, neuroscience, etc. The goal of this workshop was to present emerging problems and machine learning techniques in computational biology, with a particular emphasis on methods for computational learning from heterogeneous data.

We received 37 extended abstract submissions, from which 13 were selected for oral presentation. The current supplement contains seven papers based on a subset of the 13 extended abstracts. Submitted manuscripts were rigorously reviewed by at least two referees. The quality of each paper was evaluated with respect to its contribution to biology as well as the novelty of the machine learning methods employed.

